# A Successful Conservative Management of Twin Gestation of Complete Molar Gestation and Co-Existing Normal Fetus: A Case Report

**DOI:** 10.7759/cureus.74275

**Published:** 2024-11-22

**Authors:** Sadaf Ahmad, Dhivyambigai G R, Annapurneswari Subramanyan, Sindhuja Amalraj, Elamathi M

**Affiliations:** 1 Histopathology, Apollo Cancer Centre, Chennai, IND; 2 Obstetrics and Gynaecology, Apollo Cradle Hospitals, Chennai, IND; 3 Radiology, Apollo Cradle Hospitals, Chennai, IND; 4 Pathology, Apollo Hospitals, Chennai, IND

**Keywords:** case report, complete molar gestation, histopathology, molar pregnancy, twin gestation

## Abstract

Twin pregnancies consisting of one normal fetus and one complete mole are very rare. The main concerning risks associated with the continuation of such pregnancy are hyperthyroidism, theca lutein cysts, preeclampsia, and the development of GTD (gestational trophoblastic disease) spectrum (neoplasia) in the mother, which is due to high human chorionic gonadotropin (HCG) values, and intrauterine death and prematurity in the coexistent normal fetus. We report the successful outcome of conservative management in a healthy mother and baby. A 29-year-old patient was diagnosed with a complete mole and coexisting fetus in the 13th week of pregnancy. The risks of continuing with molar pregnancy were discussed in detail with the patient after informed consent to continue the pregnancy was obtained. With close monitoring, the pregnancy progressed, and she had spontaneous preterm labor and delivered an alive, healthy baby at 34 weeks. Post-delivery, the mother and child had no complications. Follow-up beta HCG dropped to less than 5 IU/mL at six weeks post-delivery. However, close follow-up was continued for the next six months, ensuring optimum health for the mother.

## Introduction

Molar pregnancy encompasses a benign spectrum of gestational trophoblastic disease [1]. Multiple pregnancies with a mole and a coexisting live fetus are isolated occurrences, with the incidents being one in 22,000-1,00,000 pregnancies [2]. Cytogenetic anomalies in fertilization can lead to molar pregnancy, which can be either a complete or partial mole [1]. A twin pregnancy with a complete hydatidiform mole resulting in a healthy newborn is a rare occurrence [3]. Symptoms associated with hydatidiform mole are usually vaginal bleeding, abdominal pain, passage of vesicles, nausea, vomiting, and rapid abdominal enlargement [4]. The delicate balance between managing the complications of pregnancy and protecting the life of the fetus is hard to maintain [5]. Very few twin pregnancies with hydatidiform moles continue to term gestation [2]. We hereby report a case of twin pregnancy with a hydatidiform mole and coexistent fetus, where the pregnancy was continued and the patient delivered a live baby.

## Case presentation

**Figure 1:**
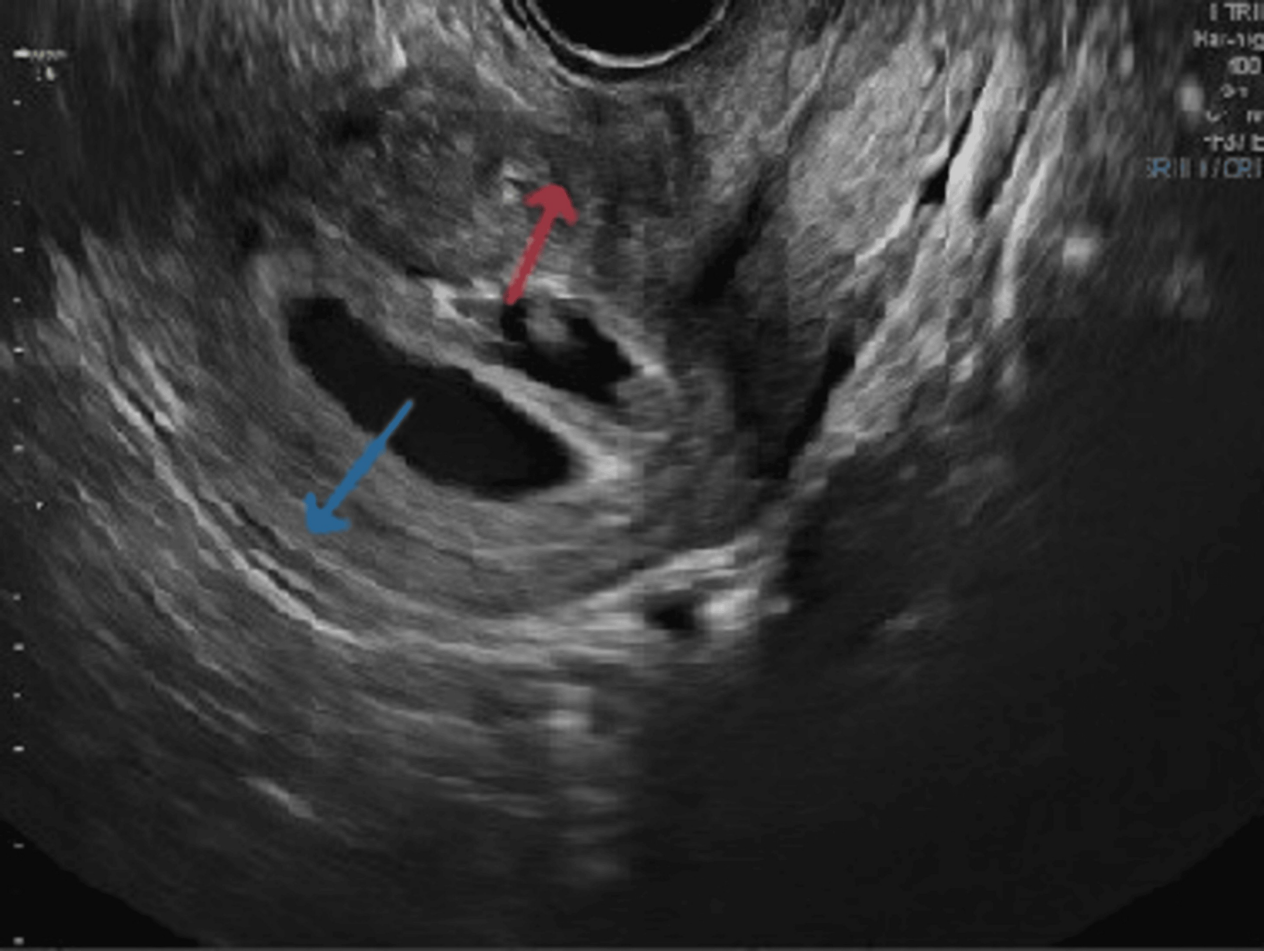
Scan at seven weeks showing twin sacs. The blue arrow indicates sac 1, and the red arrow indicates sac 2. Sac 2 is the collapsed sac, which turned out to be molar mass on NT scan at the 12th week. NT, nuchal translucency

A 29-year-old female with a previous history of two ectopic pregnancies presented to the OBG (obstetrics and gynecology) Department with a history of missed periods. In view of the history of previous ectopic pregnancies, a scan performed at five weeks showed a single intrauterine gestational sac. A follow-up scan in the seventh week showed a single live intrauterine pregnancy and a collapsed gestational sac adjacent to the live gestation (Figure 1).

**Figure 2:**
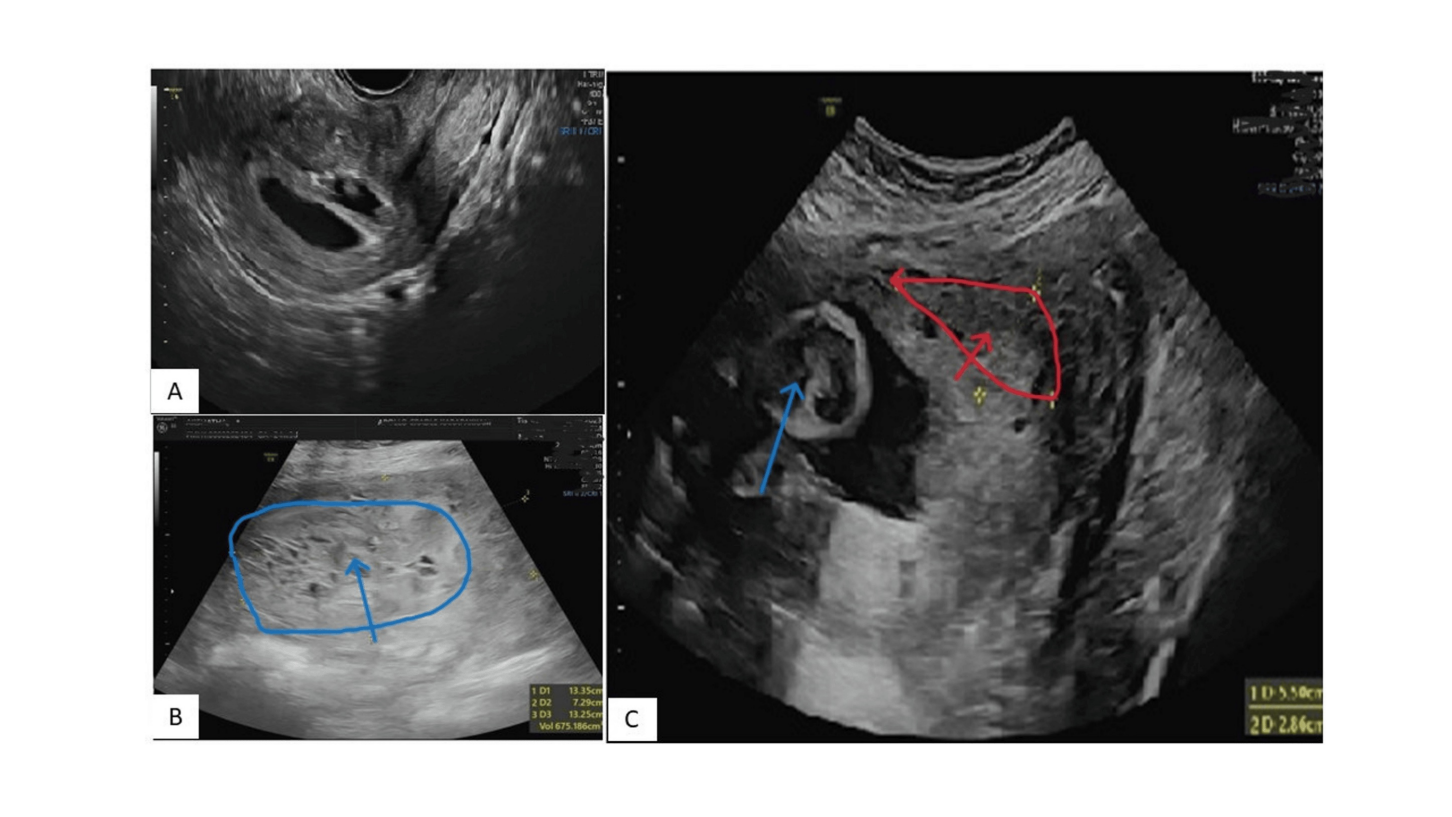
(A) Early pregnancy scan showing twin gestation sacs with fetal pole seen in one of the sacs and anembryonic second sac. (B) Molar mass with a snowstorm appearance. (C) Regressed molar mass in the third trimester. Blue arrow indicates a normal fetal sac with fetal part and liquor noted. Red arrow indicates a regressed molar mass (size reduced as compared to the size at 12 weeks).

We were considering a differential diagnosis of twin gestation sacs with a viable single fetus in one sac and a missed miscarriage of another sac. The patient did not have any complaints of spotting during the first trimester. She was called for an NT (nuchal translucency) scan at 12 weeks. The scan showed an enlargement of the collapsed sac with the typical snowstorm appearance (Figure 2).

The molar mass was occupying the lower uterine segment, covering the os and the normal sac, with a live, growing fetus seen present towards the fundus of the uterus. The beta HCG (human chorionic gonadotropin) values were found to be higher for the gestation period. At this stage, our diagnosis was of a twin with partial moles or twin gestation with a normal fetus and complete hydatidiform moles. Hence, to differentiate between a partial and complete mole, amniocentesis was performed to detect the karyotyping of the fetus. The fetus was found to have a normal karyotype. The patient was counselled after amniocentesis, explaining to her the condition of molar pregnancy with a co-existing normal fetus and the risks associated with continuing pregnancy, such as preeclampsia, thyrotoxicosis, embolism, increased perinatal morbidity due to prematurity, and placental trophoblastic disease. The patient gave informed consent to continue the pregnancy. She was kept under close follow-up with blood test monitoring and ultrasound monitoring.

The patient remained asymptomatic until 14 weeks, after which she started bleeding per vaginum on and off and had multiple episodes associated with pain until around 26 weeks. She was hospitalized twice for the management of acute bleeding episodes. During such active bleeding, she was given inj. tranexamic acid 1 g IV STAT, followed by oral medicine tablet tranexamic acid 500 mg three times a day for three days. The patient was given micronized progesterone sustained release 300 mg twice daily till 28 weeks and injection hydroxy progesterone caproate 500 mg IM weekly between 14 and 28 weeks.

Beta HCG levels showed an increasing trend from the time of diagnosis of molar gestation (12 weeks) to 26 weeks, after which HCG levels gradually started dropping, reaching the level of 20,000-30,000 at 28 weeks from 200,000 at 12 weeks. The size of the mass showed a reduction from an earlier size of 13 cm to 7 cm. During this 12- to 28-week period of monitoring, she had anemia due to multiple ongoing episodes of bleeding. The anemia was treated with intravenous iron injections. Her thyroid profile revealed thyroid-stimulating hormone (TSH) suppression but did not require any medications. Around 28 weeks, TSH values became normal as HCG values started decreasing. All her other blood tests, including liver function tests, renal function tests, urine protein, and blood pressure, were within normal limits. At the 26th week, she was given prophylactic steroid coverage in view of her anticipated preterm delivery. Her bleeding stopped after 28 weeks, and she was stable. At 34 weeks, she presented with active preterm labor. An emergency cesarean section was performed as the molar mass was in the lower uterine segment. An intraoperatively lower segment transverse incision was made at the level of uterovesical (UV) fold reflection after opening the UV fold.

**Figure 3:**
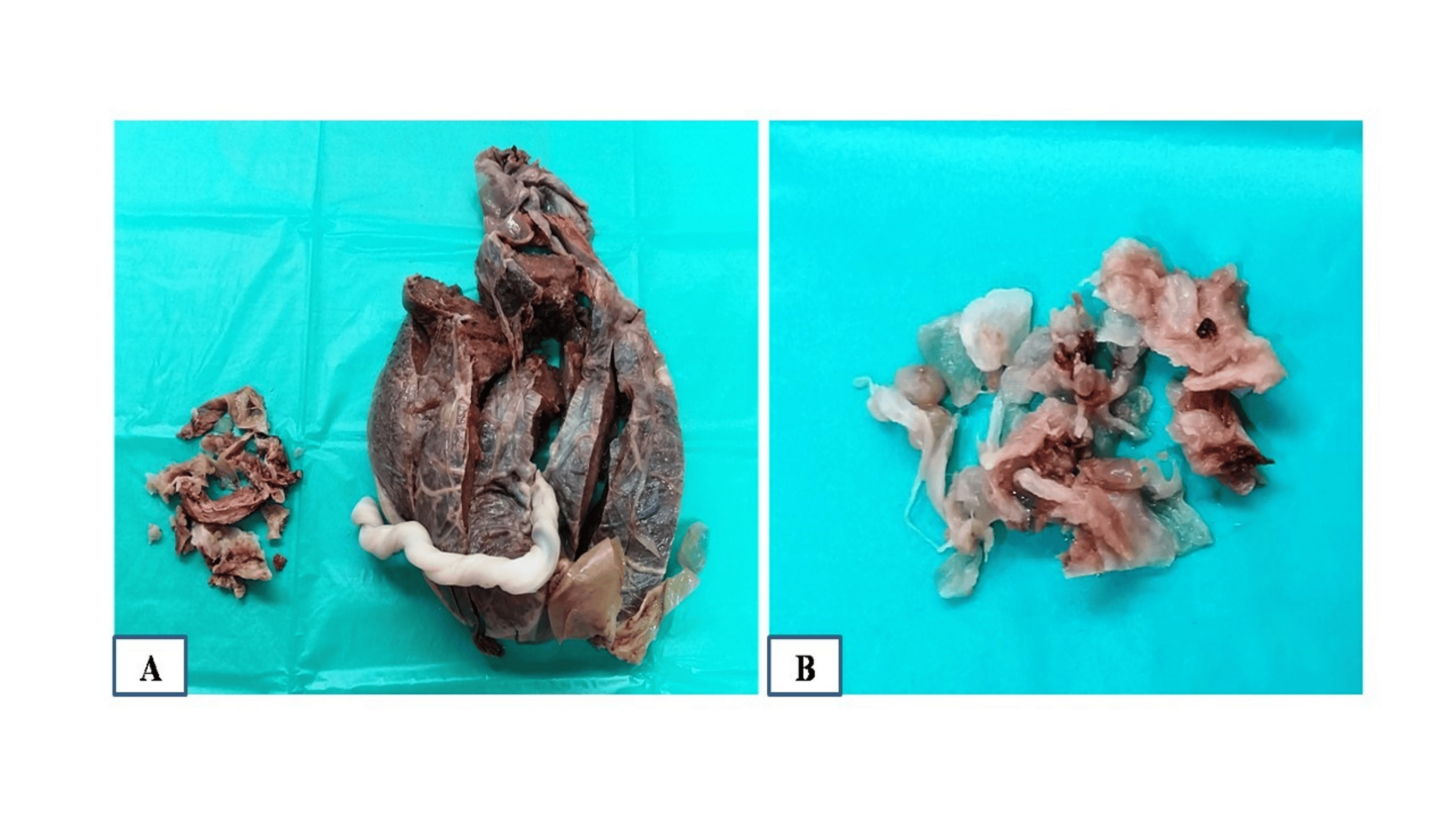
Macroscopic images. (A) Placenta with umbilical cord and (B) fragments of infarcted placental tissue with vesicles.

Since the molar mass had regressed in size, it was not interfering with baby delivery. She delivered a healthy baby boy as cephalic weighing 2.2 kg. The normal placenta with the umbilical cord and membrane of the normal baby and the molar mass in the lower segment with vesicles were sent for histopathological examination (Figure 3).

**Figure 4:**
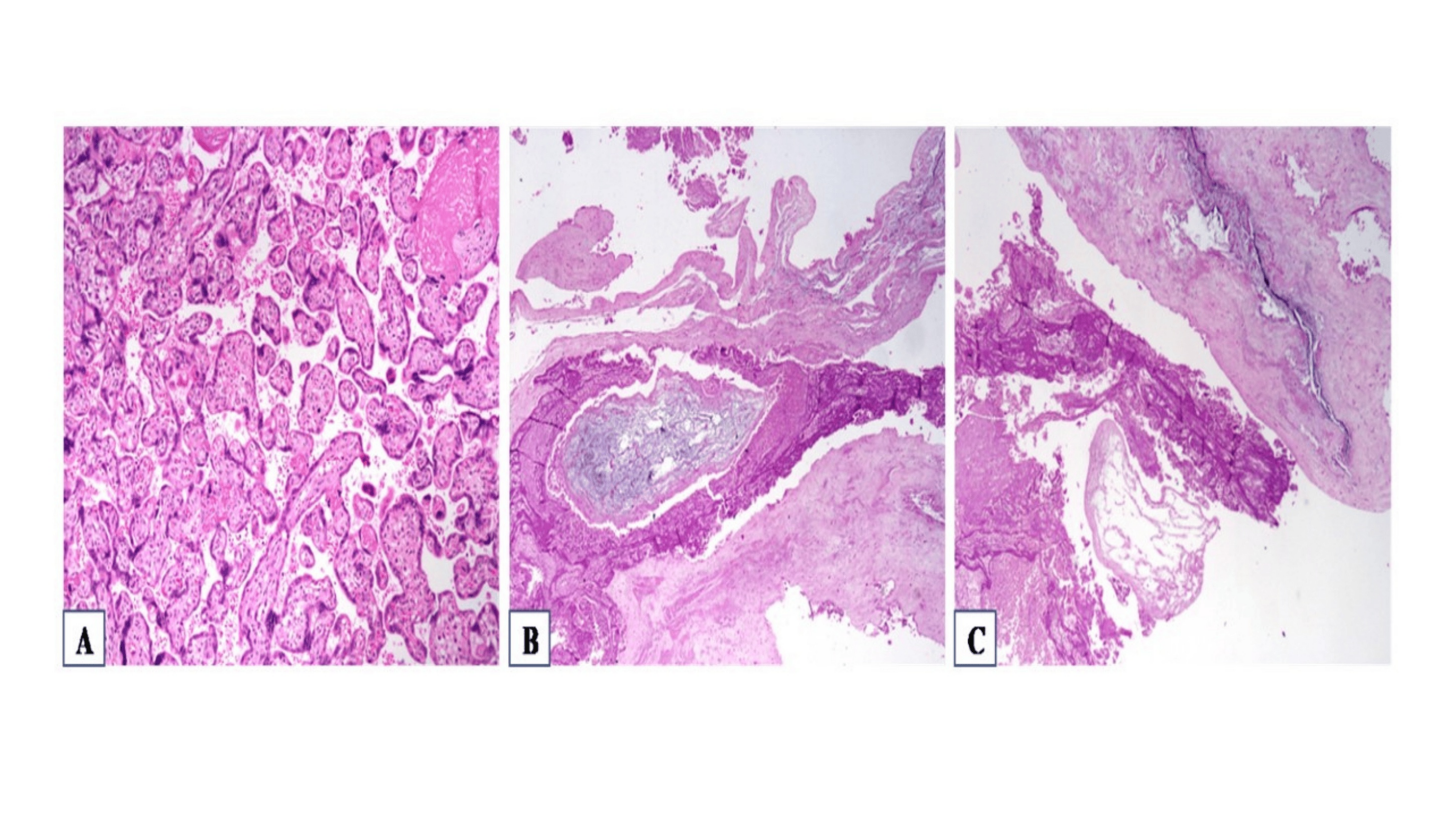
Microscopic images. (A) Normal placenta with mature villi (at 10x magnification). (B) Degenerated villi with molar change (at 4x magnification). (C) Infarcted placental tissue with ghost outline of edematous villi (at 4x magnification).

The placenta of a normal, developing baby showed no specific pathology. The other molar tissue with vesicles on histopathological examination showed infarcted, degenerated placental tissue with outlines of distended villi and a few enlarged, dilated cisterns. Infarcted trophoblastic tissue was also seen (Figure 4).

In view of all these findings, infarcted placental tissue with features suggestive of molar gestation was identified. Post-delivery, the mother and child had no complications. Follow-up beta HCG dropped to less than 5 IU/mL at six weeks post-delivery. However, close follow-up was continued for the next six months, ensuring optimum health for the mother.

## Discussion

Vassilakos et al. brought to light two pathologic entities, partial and complete hydatidiform moles, with different mechanisms of origin based on cytogenetic analysis [6]. Major risk factors that are associated with complete hydatidiform moles and partial hydatidiform moles are extreme maternal age (<15 years and > 35 years) and previous molar pregnancy [5]. The best modality for screening and detection of this condition is an ultrasound, which, if performed in the early second trimester, has a detection range of 43% to 68% [5]. Suspected or confirmed diagnosis of hydatidiform mole can be dealt with in two ways: elective termination of pregnancy or comprehensive prenatal care in a referral center for gestational trophoblastic disease [1]. The presence of a partial hydatidiform mole in a fetus is always triploid, and termination of pregnancy in such cases cannot be avoided [3]. However, in contrast, continuation of pregnancy can be considered in the case of a complete hydatidiform mole, with chances of living normal features being delivered [7]. There is an approximately 25% chance of achieving a live fetus in a normal pregnancy with a complete coexisting hydatidiform mole [8].

Sanchez Ferrer et al. stated that continuation of birth is possible in nearly 60% of cases, and termination of pregnancy is not indicated if the fetus is normal [9]. No previous reports or studies indicate that if pregnancy is kept to term, it would increase the incidence of invasive mole or choriocarcinoma [10]. However, at birth, the chances of early fetal loss are 40%, the risk of premature delivery is 36%, and the risk of preeclampsia is 20% [8].

Placental trophoblastic disease in the complete hydatidiform mole is known to be in the range of 16-50%, as compared to 14%-33% in partial moles. The placental trophoblastic disease is seen more commonly in twin molar pregnancies with maternal complications such as preeclampsia and hyperemesis [1]. Our patient did not develop any aggressive medical conditions, and the pregnancy was allowed to be continued under close surveillance.

The levels of beta HCG in a successful pregnancy with a viable fetus start reducing, and the sonography reveals a decrease in the size of the molar tissue [3]. This was seen in our case too.

The patient's condition is highly dependent on beta HCG levels, and following it up post-pregnancy is extremely vital [11]. There is a proclivity for the molar tissue to be present in the lower segment, closer to the os [2]. This was similar to that in our case. Hence, it results in on-and-off bleeding. In 15%-20% of cases of complete hydatidiform moles and less than 5% of cases of partial hydatidiform moles, there is a chance of post-molar progression to gestational trophoblastic neoplasia [4].

Hence, patients with a complete hydatidiform mole are followed for six months post-normalization of beta HCG.

## Conclusions

The occurrence of a twin pregnancy with a hydatidiform mole is a rare condition that, when detected, requires a thorough evaluation under close surveillance for an optimal outcome. In cases of twin gestation - complete hydatiform mole with co-existent fetus having normal karyotype and no gross anomalies - continuation of pregnancy can be considered if there are no maternal complications.
